# Design of DNA Storage Coding with Enhanced Constraints

**DOI:** 10.3390/e24081151

**Published:** 2022-08-19

**Authors:** Xiangjun Li, Shihua Zhou, Lewang Zou

**Affiliations:** Key Laboratory of Advanced Design and Intelligent Computing, Ministry of Education, Dalian University, Dalian 116622, China

**Keywords:** DNA storage, Aquila Optimizer, coding enhancement constraint, DNA coding design

## Abstract

Traditional storage media have been gradually unable to meet the needs of data storage around the world, and one solution to this problem is DNA storage. However, it is easy to make errors in the subsequent sequencing reading process of DNA storage coding. To reduces error rates, a method to enhance the robustness of the DNA storage coding set is proposed. Firstly, to reduce the likelihood of secondary structure in DNA coding sets, a repeat tandem sequence constraint is proposed. An improved DTW distance constraint is proposed to address the issue that the traditional distance constraint cannot accurately evaluate non-specific hybridization between DNA sequences. Secondly, an algorithm that combines random opposition-based learning and eddy jump strategy with Aquila Optimizer (AO) is proposed in this paper, which is called ROEAO. Finally, the ROEAO algorithm is used to construct the coding sets with traditional constraints and enhanced constraints, respectively. The quality of the two coding sets is evaluated by the test of the number of issuing card structures and the temperature stability of melting; the data show that the coding set constructed with ROEAO under enhanced constraints can obtain a larger lower bound while improving the coding quality.

## 1. Introduction

Storage is essential for the preservation of history and the dissemination of knowledge. With time, the data required to be stored is increasing, and the storage mode has undergone great changes. From the ancient knot storage of digital records to paper records to CD and hard disk storage technology, the storage density has also made a qualitative leap. In today’s big data era, the increasingly mature informatization in all walks of life has brought about not only the improvement of productivity but also the explosive growth of data volume. Soon, mainstream storage media may be unable to carry massive amounts of information. At the same time, the short storage life of the existing storage media will lead to high maintenance costs. Therefore, the discovery of new storage media is imminent [1]. DNA can reach $10^{19}\;\mathrm{bit}/{\mathrm{cm}}^{3}$, which is $10^{6}$ times that of hard disk in terms of storage density. In addition, as genetic material, DNA can ensure the accurate reproduction and inheritance of life, and samples of tens of thousands of years can still be restored to complete DNA fragments, indicating that it has strong stability. Therefore, in the mid-1960s, Neiman had a preliminary discussion on the concept of gene memory [2]. However, at that time, DNA sequencing and synthesis technology had just started, which technically limited the development of DNA storage. Thus, DNA data storage could not be realized. Davis [3] encodes DNA according to the molecular size of bases and successfully stores abiotic information in DNA. However, due to the defects in decoding, the original date cannot be accurately reconstructed.

In order to achieve efficient DNA storage, it is necessary to solve the deletion, insertion, replacement, and other errors that are prone to occur in the decoding process, which requires the use of coding methods suitable for DNA storage to construct high-quality DNA storage sets. To solve this problem, predecessors have made many explorations. To build a more reasonable DNA sequence library, Garzon et al. [4] proposed the corresponding combinatorial constraints during coding in 2004. In 2009, Ailenberg [5] applied an improved Huffman coding method to the plasmid-based DNA repository, making it have efficient and reliable information retrieval and assembly functions. In 2012, Church [6] and others proposed a method for storing DNA that encodes information in various ways to avoid errors when reading encoded DNA sequences. One year later, Goldman [7] and others proposed a DNA storage scheme that can restore 100% of the original information, storing a larger amount of information than before. In 2015, Grass et al. [8] translated 83 kB of information into 4991 DNA fragments and used error correction codes to correct errors in stored procedures. This study shows that information can be in long-term storage and accurately recovered. In 2016, Hong [9] et al. used algebraic number theory to construct DNA coding sets with larger length, more quantity, and GC content. In the same year, Blawat [10] and others developed a forward error correction scheme, which is powerful and efficient, and completed error-free storage and retrieval of 22 MB of data. Bornhol et al. [11] applied the key–value storage pattern to DNA storage systems and further formed a new coding scheme, which can provide controllable redundancy and make a trade-off between reliability and density. In 2017, Gabry et al. [12] constructed a series of DNA storage codes with asymmetric Lee distance on the basis of a quaternary alphabet, which can better overcome the errors such as pairing and replacement in coding. Erlich et al. [13] proposed a new coding method of DNA fountain; this method’s code density is close to what is theoretically optimal in this scheme, and the performance of retrieving experimental data is improved by order of magnitude compared with previous work. In 2018, Yazdi [14] introduced the concept of weak cross-correlation (WMU) sequence, in which the prefix and suffix of the coding are constrained and the Hamming distance is combined with the constraint set to avoid the production of homopolymers in the primer sequence. Organick et al. [15] stored up to 200 MB of data in DNA molecules, realized random access in large-scale systems, and tried to use single molecule sequencing (SMS) to read and recover data. Nguyen et al. [16] stored 2046 words in the plasmid-based text and proved through experiments that DNA storage is reliable even after a long period of preservation. In the same year, the Limbachiya team [17] obtained the lower bound of the coding sets stored in DNA through an altruistic algorithm under more stringent constraints, which better limited the generation of homopolymers. Song [18] established a mapping rule between binary coding and DNA sequences, and this rule requires DNA sequences to satisfy run-length and GC-content constraints. In this method, each wrong nucleotide will lead to a wrong sequence with a length of 2n. The error rate is much lower than that in the DNA fountain method, and the rate of 1.9 bits/nt is achieved, which effectively reduces the error propagation. In 2019, Choi [19] et al. realized the information capacity of 3.37 bits/character by using degenerate bases other than A, T, G, and C as coding characters, which can reach twice the highest information capacity in the past. Zhang et al. [20] used quaternion Huffman coding to compress original files to increase storage density and successfully encoded and decoded 5.2 kB files using low redundancy quaternion Hamming codes. Anavy et al. [21] found a DNA synthesis method that can significantly shorten the synthesis cycle. The core of the method is to mix nucleotides in a predetermined proportion. In order to ensure local and global stability while satisfying biochemical constraints, Wang team [22] proposed a coding construction method, which has a high bit rate and low coding complexity. There is a limit on the writing size of DNA molecules in DNA storage. In order to address this issue, Heckel et al. [23] quantitatively analyzed the lost and erroneous molecules in DNA data storage, which provides a new idea to remove this limitation.

With the substantial reduction of the cost of DNA sequencing, the encoded information density is gradually approaching the theoretical value, and the goal of making DNA storage a commercial storage method is about to be achieved. However, the problem of low sequencing accuracy still exists. Aiming at the insertion, deletion, and replacement errors that easily occur in stored procedures, the Press team [24] introduced the coding technology of the HEDGES error correction code in 2020. In 2021, Yin [25] proposed a new MPA algorithm, called QRSS-MPA, which can obtain high-quality and larger DNA coding sets, effectively reduce the error rate, and improve storage efficiency. Organick [26] studied the preservation method of synthetic DNA based on its short length and discussed the trade-off between stability and density. In 2022, in order to adapt to the characteristics of DNA synthesis and sequencing, Ren et al. [27] developed two highly reliable coding systems (RALR and RABR) suitable for tetranucleotide, hexanucleotide, and octanucleotide. The average coding efficiency reached 1.27 bits/nt, 1.61 bits/nt, and 1.85 bits/nt, respectively, without arithmetic compression but with error correction.

In order to reduce reading errors caused by low-quality DNA coding, it is necessary to improve the quantity and quality of coding sets. To keep the number of secondary structures as low as possible in the DNA coding sets, the repeated tandem sequence constraint is proposed in this research. To resolve the issue that the traditional distance constraint is not accurate in evaluating the overall similarity between sequences and cannot effectively limit the non-specific hybridization reaction between them, an improved DTW distance constraint is proposed in this research. In addition, based on the original algorithm of AO, the ROEAO algorithm is proposed. Combined with GC-content, No-runlength, and Hamming distance constraints, a DNA storage coding set with different lengths and specific constraints is constructed. This coding set has a certain error correction ability, but its nature is not very stable. In order to construct a higher-quality coding set, the ROEAO algorithm is used to construct the coding set with two enhanced constraints. Finally, in order to evaluate the stability of this coding set, this study compares the hairpin structure and melting temperature of the subset with different lengths and different distances. The results from the test show that the robustness of the DNA storage coding set is effectively improved under the two enhanced constraints.

## 2. Constraints on DNA Codes

### 2.1. Traditional Constraints

#### 2.1.1. GC-Content Constraint

The four basic bases of DNA sequence are A, T, C, and G. Its *GC* content determines the thermal stability of the sequence [28]. For DNA sequences with length *n*, the *GC* content is calculated as follows:

(1) $$GC(n)=\frac{\left|G|+|C\right|}{\left|n\right|}$$

#### 2.1.2. Hamming Distance Constraint

The probability of non-specific hybridization between DNA sequences is proportional to their similarity. In order to prevent the occurrence of non-specific hybridization in the coding process, a condition is required to restrict their similarity. In this research, Hamming distance is considered to be used to constrain the coding. For two DNA sequences *u*, *v*, the XOR of the *i*-th base is represented by *h(u_i_,v_i_)*, and the calculation formula of Hamming distance *H(u,v)* is:

(2) $$H(u,v)=\sum_{i=1}^{n}h\left(u_{i},v_{i}\right),h\left(u_{i},v_{i}\right)=\{\begin{array}{l} 0,u_{i}=v_{i}\\ 1,u_{i}\neq v_{i} \end{array}$$

#### 2.1.3. No-Runlength Constraint

When constructing the coding of DNA storage, it is required that no same consecutive base is allowed at any adjacent position. Otherwise, it is easy to cause errors in the process of sequencing and synthesis. Therefore, in the coding process, constraints should be used to avoid such homopolymers. Such constraint is called no No-runlength constraint (NL). For the sequence *u(u*_1_, *u*_2_, *u*_3_,…, *u_n_)* with length *n*, the constraint is defined as follows:

(3) $$u_{i}\neq u_{i-1},i\in [1,n]$$

### 2.2. Enhanced Constraints

#### 2.2.1. Repeated Tandem Sequence Constraint

**Figure 1:**
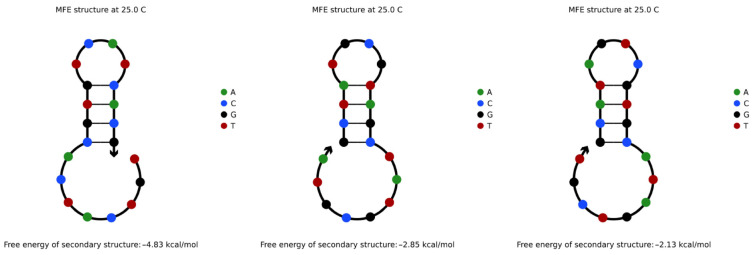
Secondary structure formed by sequence repeat tandem.

When encoding information, the repeated occurrence of some information will cause the coding sequence to repeat continuously. In this research, such a sequence structure is called a repeated tandem sequence. The repeated tandem sequence is easy to fold back to form a secondary structure similar to that shown in Figure 1 due to some special base arrangement [29]. The three sequences are the repeated tandem sequences of TGTCATCACG, GCTATGCGTA, and GCATAGTCGT, respectively. However, random reading in the DNA storage process is realized by PCR amplification reaction [30]. In the PCR amplification reaction, if the single strand of the amplified template needs to be folded back, the structure formed by folding back is likely to compete with the combination of primer and template. Thus, high-efficiency reading cannot be realized [15].

Figure 1 is the structure analysis diagram obtained from the NUPACK simulation experiment. It proves that, in the two same subsequences *L*: TGTCATCACG and *L*′: TGTCATCACG of the repeated tandem sequence TGTCATCACGTGTCATCACG, the base fragment CG of *L* and the base fragment TG of *L*′ form a base combination of CGTG. This combination easily complements the inverse sequence of the CACG fragment in *L*′, resulting in a hairpin structure that is not conducive to PCR amplification and reading. In the course of multiple experiments, it was found that, when the base pairs of such complementary fragments in the repeated tandem sequence reached three or more pairs, the sequence was easy to fold back and self-complement to form the above secondary structure.

**Figure 2:**
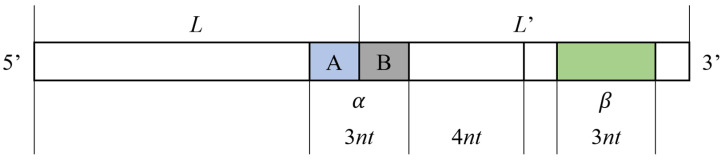
Repeat tandem structure.

Therefore, in order to avoid secondary structure when the coding sequence appears continuously, the concept of repeated tandem sequence constraint (RTSC) is proposed in this research. For a sequence *L*(*l*_1_, *l*_2_, *l*_3_…, *l_n_*) with length N, the constraint is defined as follows: for two identical sequences *L* and *L*′, when they appear continuously in series, as shown in Figure 2, the three-base combination formed by the tail *A* of *L* and the head *B* of *L*′ is recorded as *α*; the formula is as follows:

(4) $$A=\{\begin{array}{c} l_{n},i=1\\ (l_{n}{}_{-1},l_{n}),i=2 \end{array},B=\{\begin{array}{c} ({l^{\prime }}_{1},{l^{\prime }}_{2}),i=1\\ {l^{\prime }}_{1},i=2 \end{array},\alpha =\left[\begin{array}{cc} A_{i=1} & B_{i=1}\\ A_{i=2} & B_{i=2} \end{array}\right]$$

In the remaining sequence, in which the head *B* and its last four bases (the ring region used to form the hairpin structure) are removed from *L*′, any combination of three consecutive bases is recorded as *β*; the formula is as follows:

(5) $$\beta =\left[\begin{array}{lll} l_{n-2} & l_{n-1} & l_{n}\\ l_{n-3} & l_{n-2} & l_{n-1}\\ \vdots & \vdots & \vdots \\ l_{8-i} & l_{9-i} & l_{10-i} \end{array}\right]$$

If any row of *α* is complementary to the reverse sequence of any row of *β*, the above-mentioned secondary structure is easily formed; thus, the sequence that meets the constraint of repeated tandem sequence should meet the following conditions:

(6) $$\forall i\in [1,2],j=\{\begin{array}{c} {}[1,n-8],i=1\\ {}[1,n-7],i=2 \end{array},s.\;t.\;\bar{\alpha }(i,:)\neq \tau (\beta (j,:))$$

**Figure 3:**
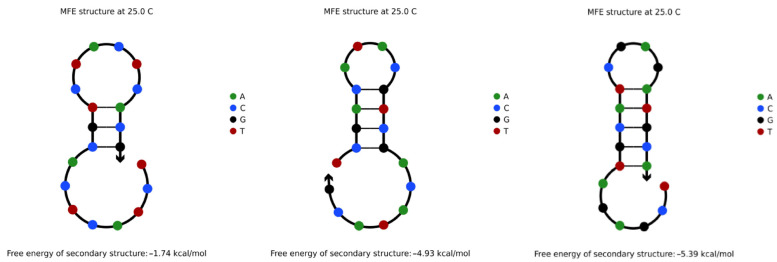
Sequences that do not satisfy RTSC constraints.

The DNA sequences that do not satisfy the RTSC constraints were screened out by MATLAB experiments, and NUPACK simulation experiments were performed on these sequences. The results show that the repeated tandem of these sequences will indeed generate such secondary structures, as shown in Figure 3:

#### 2.2.2. Improved DTW Distance Constraint

**Figure 4:**
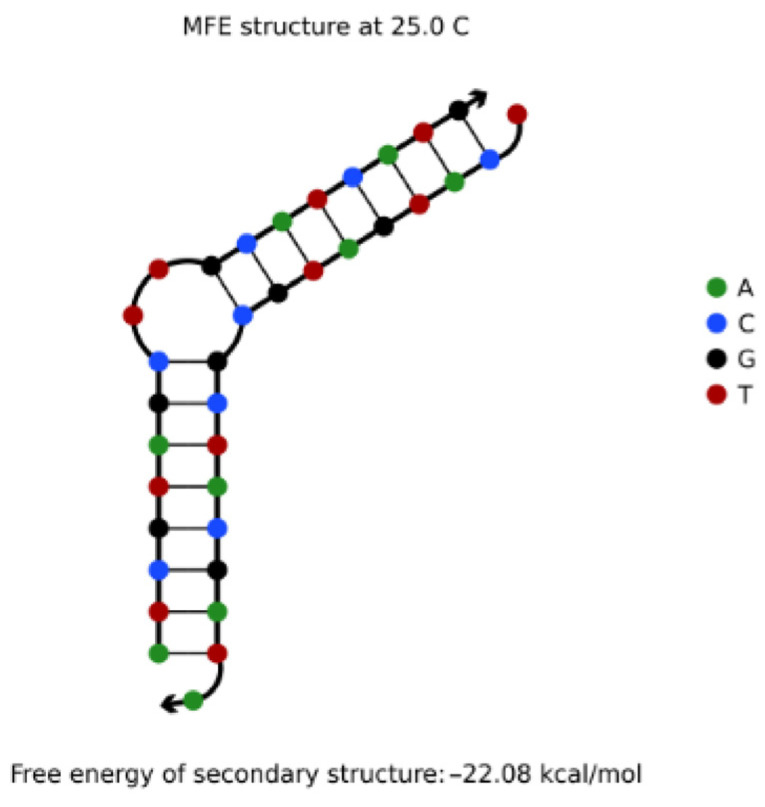
Sequence A and B hybridize to form the secondary structure of uplift.

The NUPACK tool was used to simulate two DNA sequences A: ATCGTAGCTTGCATCATG (5′→3′) and B: TCATGATGCGCTACGATA (5′→3′) with a concentration of 1 μΜ. It was found that the reaction products of 1 μΜ were the secondary structure of the uplift shown in Figure 4. Obviously, the non-specific hybridization reaction can easily occur between the two sequences.

For two DNA sequences *L*_1_, *L*_2_, if there is enough “similarity” between sequence *L*_1_ and the complementary sequence *L*_2_′ of sequence *L*_2_, they are prone to non-specific hybridization under appropriate conditions, resulting in secondary structures such as uplift, shift pairing, and so on, thus reducing the efficiency of reading sequences in DNA storage. For example, the complementary order of B in the direction of 3′ to 5′ is listed as TATCGTAGCGCATCATGA, which is very similar to A: ATCGTAGCTTGCATCATG as a whole. However, if the traditional distance index is used to judge the similarity between A and B′, the probability of non-specific hybridization between A and B′ may be underestimated because the value is too large (for example, Hamming distance = 18). Therefore, in order to better limit the non-specific hybridization of this kind of sequence, it is necessary to use more flexible distance indicators.

Dynamic Time Warping (DTW) [31] is mainly proposed for sequence matching to find similarity. The DTW algorithm scales the series through warping distortion and then calculates the minimum distance between the two time series to get the maximum similarity between them. Traditional DTW generally uses Euclidean distance to calculate the shortest path, but, to judge the non-specific hybridization of DNA sequence, one only needs to consider whether the bases match. Therefore, the Hamming distance, which is used to represent the exclusive or relation, is used instead of the Euclidean distance. Consequently, the shorter the improved DTW distance between sequence *L*_1_ and the complementary sequence *L*_2_′ of *L*_2_, the higher the similarity between *L*_1_ and *L*_2_′; that is, the non-specific hybridization reaction is more likely to occur between *L*_1_ and *L*_2_. In order to express the distance between *L*_1_ and *L*_2_ as computing the distance between *L*_1_ and *L*_2_′, *L*_1_ and *L*_2_ are represented as the following time series:

(7) $$L_{1}=[l_{1},l_{2},\ldots ,l_{i},\ldots l_{n}]=\{\begin{array}{c} 0,l_{i}=A\\ 1,l_{i}=T\\ 2,l_{i}=C\\ 3,l_{i}=G \end{array}\;,\;L_{2}=[l_{1},l_{2},\ldots ,l_{j},\ldots l_{m}]=\{\begin{array}{c} 0,l_{j}=T\\ 1,l_{j}=A\\ 2,l_{j}=G\\ 3,l_{j}=C \end{array}$$

For two DNA sequences *L*_1_, *L*_2_, the improved DTW distance calculation formula between them is as follows:

(8) $$\begin{array}{cc} d_{DTW}(L_{1},L_{2})= & \{\begin{array}{c} 0,\;if\;L_{1}=0\;and\;L_{2}=0\\ \infty ,\;if\;L_{1}=0\;or\;L_{2}=0\\ d_{DTW}(H(L_{1}),H(L_{2}))+\min\{\begin{array}{l} d_{DTW}((L_{1}),R(L_{2}))\\ d_{DTW}(R(L_{1}),L_{2}),other\\ d_{DTW}(R(L_{1}),R(L_{2})) \end{array} \end{array} \end{array}$$

*H (L)* represents the first base of the DNA sequence, *R (L)* denotes the subsequence except for the first base in the sequence, and *d_DTW_(L_i_,L_j_)* represents the Hamming distance between *L_i_* and *L_j_.*

**Figure 5:**
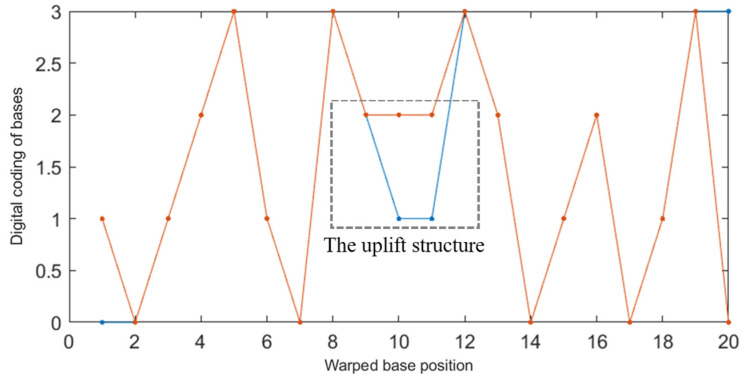
DTW analysis image of sequence A and B.

The improved DTW algorithm was used to calculate the shortest distance between A: ATCGTAGCTTGCATCATG (5′→3′) and B: TCATGATGCGCTACGATA (5′→3′). The result was d_DTW_ = 4, which is much smaller than the traditional Hamming distance (*d* = 18). In addition, it can be seen from the DTW images of the two sequences (the red line in Figure 5 indicates a and the blue indicates b) that the improved DTW algorithm can well predict the bulge structure generated between DNA sequences, which is consistent with the results of NUPACK simulation experiments. Therefore, using the improved DTW distance to constrain the DNA storage coding can more accurately limit the possibility of non-specific hybridization between two sequences, thus improving the efficiency of the DNA coding reading phase.

### 2.3. Fitness Function

The design of DNA coding needs to follow a fitness function, where the fitness function based on traditional constraints is represented by Formula (9), and the fitness function based on enhanced constraints is represented by Formula (10), wherein *u* and *v* represent two DNA sequences of length *n*.

(9) F(n)=H(u,v) Subject to the constraints: GC-content=50%, No-runlength

(10) $$F(n)=d_{DTW}(u,v)\;\mathrm{Subject}\;\mathrm{to}\;\mathrm{the}\;\mathrm{constraints}:\;\mathrm{GC}-\mathrm{content}=50\%,\;\mathrm{No}-\mathrm{runlength},\;\mathrm{RTSC}$$

## 3. Algorithm Description

An effective model that stores a large amount of data in a small amount of DNA is essential to improve storage efficiency; that is, the model can construct a larger number of coding sets within a certain base length, so this paper proposes an improved Aquila Optimizer algorithm. The algorithm can well jump out of the local optimum so as to search for codes that satisfy the constraints in a larger range.

### 3.1. Aquila Optimizer

In 2021, Abulaliga et al. [32] proposed a new swarm intelligence algorithm called Aquila Optimizer (AO). Aquila’s hunting method is flexible and can adopt corresponding hunting methods according to the behavior of different prey. It mainly uses four hunting methods: bending vertically and flying high to select the search space, in divergent searching space through contour flight of short gliding attack to explore, in convergent search space through the low-altitude flight of slow descent attack to develop, and swooping and catching prey on foot. If $t\leq \frac{2}{3}T$ (the maximum number of iterations and the current iteration are represented by T and t, respectively), the AO algorithm switches from exploration mode to development mode. The specific mathematical expressions of the four hunting behaviors are as follows:

(11) $$X_{1}(t+1)=X_{best}(t)\times (1-\frac{t}{T})+[X_{M}(t)-X_{best}(t)\times r]\;$$

(12) $$X_{2}(t+1)=X_{best}(t)\times Levy(D)+X_{R}(t)+(y-x)\times r$$

(13) $$X_{3}(t+1)=[X_{best}(t)-X_{M}(t)]\times \alpha -r+[(UB-LB)\times r+LB]\times \delta$$

(14) $$X_{4}(t+1)=QF\times X_{best}(t)-[G_{1}\times X(t)\times r]-G_{2}\times Levy(D)+r\times G_{1}$$

where *X_best_(t), X_M_(t)*, and *X_R_(t)* represent the best position obtained so far by Aquila, the current average position in the current iteration, and the random Aquila’s position, respectively. *D* is the size of dimension, the Levy flight function is represented by *Levy(D)*, *x* and *y* describe the trajectory of Aquila during the search, and *r* and *G_1_* are random numbers from 0 to 1. *QF*, *α*, and *δ* are fixed parameters. *G_2_* is the slope of the flight when moving by Aquila.

### 3.2. The Improved Algorithm

The hunting behavior of AO in the exploration phase is aimed at simulating the fast- moving prey. Therefore, the AO algorithm has the characteristics of strong randomness and fast convergence at this stage. In Aquila’s second hunting mode, Aquila’s flight behavior is spiral. Although this spiral flight provides the AO algorithm with greater search coverage and the ability to search around the prey, this mode will make Aquila search many times in each local region. Therefore, it is easy to update the local optimal individual as the next generation individual. Because the update of Aquila’s position always depends on the current optimal individual, the result of the next iteration is also close to the local region. Eventually, the update ends at the local optimal value. In the final stage, the local development uses Levy flight because of its small search step, so the local development will fall into the local optimization because of the incomplete global search.

Therefore, the overall optimization performance of the AO algorithm is improved in this research. In order to speed up the convergence speed and enhance the ability to jump out of the local optimum while retaining the global search ability of the algorithm. Two improvement directions are considered to make up for the shortcomings of the algorithm: one is to adjust the search direction, and the other is to adjust the search step size.

#### 3.2.1. Random Opposition-Based Learning

Due to the spiral search, the individual may be close to the globally optimal region or the locally optimal region. When entering the latter region, it is easy to make the AO stay at the local optimal. In order to leave the non-global optimal region, the direction of individual updates needs to be changed in time. In the development stage, the use of opposition-based learning [33] can provide a new direction for the update of individuals, thereby increasing the probability of individuals entering the global optimum and expanding the search space for individuals. The principle of opposition-based learning [33] is to produce a completely reversed solution according to the current solution, but when neither of these two individuals is close to the optimal region, it cannot effectively achieve the purpose of this improved scheme, and even an individual far from the global optimal solution may be generated. Therefore, in order to improve the efficiency of reverse learning and prevent the global search phase from ending prematurely, this research adopts random reverse learning, which is more random [34].

(15) $$\bar{X}=a+b-rand\times X$$

Among them, *a* and *b* are the lower and upper bounds of the problem, respectively, the rand is a random number from 0 to 1, X∈[a,b], and the random opposition solution of ?X is X. This strategy generates a set of solutions that are not wholly opposed to the current solution, which can change the direction and jump out of the local optimal and effectively deal with the above extreme cases.

#### 3.2.2. Eddy Jump

The step size of the individual position movement is very critical to the optimization of the algorithm. If the same step size is maintained, it is easy to jump over the global optimal region or be unable to reach the global optimal region before the end of the update. In the process of AO updating individual positions, because the step length of Levy flight is short, the updating effect is relatively weak, and it is difficult to escape from the local optimal area, so a scheme with a longer moving step length is needed. In the Marine Predators Algorithm [35], eddy usually changes the foraging trajectory of marine predators. In order to avoid this eddy, marine predators usually use a long jump to avoid local optimization stagnation. At the same time, the step size of such jumps has certain randomness and can balance search and local development. Inspired by this, this large step jump is used to improve the development stage of AO to avoid optimization stagnation.

(16) $$X=X+(F\times (1-r)+r)\times (X_{i}-X_{j})$$

where *F* equals 0.2, *X_i_* and *X_j_* are two random solution vectors of *X*, and *r* is a random number from 0 to 1.

The AO algorithm that combines the above two strategies is called the ROEAO algorithm.

#### 3.2.3. ROEAO Algorithm Description

Algorithm 1 shows the pseudo-code of the ROEAO algorithm.
**Algorithm 1** ROEAO algorithm pseudo code.Set a series of initial parametersRandomly set the initial individual *Xi*(*i* = 1,2,…,N) While (*t*
≤
*T*) Compute the fitness value and update *X_best_*  **for** I from 1 to N  update parameters and *X_M_(t)*   **if**
$t\leq \left(\frac{2}{3}\right)*T$ **if** rand ≤ 0.5 update the position with Equation (11) and *X_best_* **else** update the position with Equation (12) and *X_best_* **end if** **else** **if** rand ≤ 0.5 update the position with Equation (13) and *X_best_* **else** update the position with Equation (14) and *X_best_* **end if**   **end if**  **end for** Execute Random Opposition-Based Learning based on Equation (15) Execute Eddy Jump based on Equation (16) **end while** **Return** the best solution *X_best_*

### 3.3. Experiment Environment and Symbol

We ran various simulation experiments with MATLAB 2018 on Intel Core i73.6-GHz CPU. Table 1 explains the meaning of each superscript in this research. Bold data in the remaining tables indicate better results.

### 3.4. Benchmark Function Comparison

Benchmark function is a simulation of practical problems; this research used mainstream benchmark function to verify the performance of the ROEAO algorithm on different functions [36]. In this research, a total of 13 benchmark functions, including unimodal and multimodal functions, are selected to show the test results. This series of test functions contains most types of optimization problems. Thus, these test functions are well representative. The effectiveness of this algorithm is verified by comparing the ROEAO algorithm with the original algorithm AO [32] and some other classical swarm intelligence algorithms, among which Particle Swarm Optimization (PSO) [37] is a long-tested and widely used algorithm. Differential Evolution (DE) [38] is a stochastic model that simulates biological evolution. Through repeated iterations, individuals who are adapted to the environment are preserved. Grey Wolf Optimizer (GWO) [39] and Whale Optimization Algorithm (WOA) [40] are classic meta-heuristic algorithms, Marine Predators Algorithm (MPA) [35] and Harris Hawks Optimization (HHO) [41] are excellent meta-heuristic algorithms proposed in the past three years. In this round of experiments, the parameters of each algorithm were set: the dimension of the non-fixed dimension test function is 30, the maximum number of iterations is 500, and the population size of each algorithm is 30.

Unimodal benchmark function (F1–F7) tests the basic development ability of the main algorithms [42]. It can be seen from Table 2 that the standard deviation and accuracy of ROEAO were obviously better than those of other algorithms. Not only did the mean and variance results of function F1~F4 reach the optimal value but also the accuracy of F5 and F6 were higher than that of other algorithms by 12 to 33 orders of magnitude; in addition, its stability performance was much better than other algorithms. Thus, this shows that ROEAO’s benchmark development capability is stronger. Because a great many local optimal solutions exist in multimodal functions (F8~F13), the algorithm that can perform well in this kind of function can better prove its superiority in development ability [35]. In the multi-peak function test, such as Table 3, the standard deviation and average value of F9~F11 reached the optimal value. At the same time, the accuracy and stability of F12 and F13 were much better than other algorithms. In particular, it is 24~37 orders of magnitude better than other algorithms in terms of average value, which can better reflect the good performance of ROEAO jumping out of the local optimization and the fast speed of optimization.

Wilcoxon signed rank test [43] is used for statistical analysis of the above comparison results. The results are shown in Table 4, where *s/e/w* indicates that ROEAO is superior to, equal to, and worse than the algorithm used for comparison. The *p*-values of these seven results are all less than 0.05, which indicates that ROEAO is significantly different from other algorithms, and the number of functions in which ROEAO is dominant is 69~100%. It can be seen that ROEAO is obviously superior to other algorithms.

**Figure 6:**
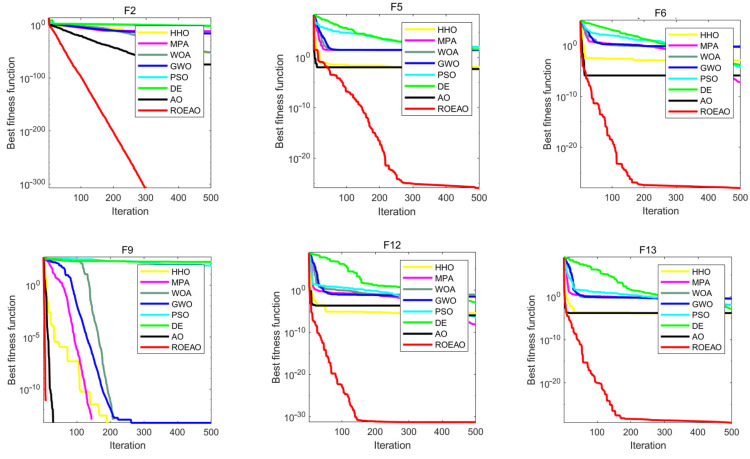
Convergence iteration curves of several benchmarks.

Showing the convergence of each algorithm as a visual image can compare their optimization performance more intuitively. In Figure 6, several convergence curves of AO, HHO, MPA, WOA, GWO, PSO, DE, and ROEAO were given. From the visual image, the ability to jump out of the local optimum and the convergence speed can be directly compared; it can be seen that these two capabilities of ROEAO have great advantages over other algorithms. In the graph in F2, ROEAO had always maintained an efficient development state. In F5 and F6, ROEAO demonstrated a strong exploration ability, the convergence speed was extremely fast, and it did not fall into local optimum, which may be affected by the strategy of increasing the search step size. In F9, it converges significantly faster than other algorithms. In F12 and F13, it was observed that ROEAO still maintains an efficient development state when other algorithms have fallen into local optimization. Generally speaking, the convergence and speed of ROEAO have always been among the best algorithms.

## 4. Experimental Results and Analysis

### 4.1. Lower Bound of Coding Set with Traditional Constraints

In order to improve the storage efficiency, it is necessary to construct a larger number of codes that meet the constraints. This paper combines traditional constraints with the ROEAO algorithm with excellent performance to construct a large-scale DNA storage code set design model. The model uses the traditional combinatorial-constrained objective function, Equation (9), as the fitness function and encodes the letters of each gene with quaternions, with 0, 1, 2, and 3 representing the bases T, C, G, and A, respectively. The specific steps of the model construction and coding are as follows:

Step 1: Initialize the parameters required by ROEAO algorithm, for example, the maximum count of iterations, the number of populations, etc.;

Step 2: Randomly generate a set of DNA sequences, and put the sequences that meet the combinatorial constraints into the initial sequence set as the first candidate sequence set to start updating;

Step 3: Start using Aquila’s four hunting methods to calculate the fitness of candidate sequences, and update the candidate sequence set;

Step 4: Perform random reverse learning on the updated set of candidate sequences to calculate the fitness of the candidate sequences, and then replace the sequences that meet the constraints of sorting according to fitness, and then replace the poor sequences in the original set;

Step 5: Perform a vortex jump operation on the candidate sequence set. The sequences that meet the combinatorial constraints are added to the candidate set;

Step 6: Judging the termination condition, if the count of iterations reaches the maximum value, the result is calculated and summarized, and the output sequence set and the maximum number of sequences are output. Otherwise, return to step 3.

The set of DNA sequences satisfying the No-runlength constraint, GC-content constraint, sequence length equal to n, and Hamming distance equal to d is represented by S^GC,NL^(n,d). In order to prove that the coding set constructed by the ROEAO algorithm can effectively reduce the errors in actual storage, this research compared it with the lower bound of 4 ≤ n ≤ 10 and 3 ≤ d < n in the coding results of the EORS algorithm [44] and altruistic algorithm of Limbachiya [17]. As shown in Table 5, ROEAO can obtain the optimal DNA coding set compared with previous work. For example, when n = 6 and d = 4, the code set constructed by ROEAO is 69% and 28% larger than the previous results, respectively.

The encoding rate is one of the factors that determines the efficiency of DNA storage, and the encoding rate depends on the number of encoding sets and the length of the encoding. The calculation formula is $R=\frac{1}{n}\log_{4}M$, where n is the length of the DNA sequence and M is the DNA code number of sets. For example, in the study of Limbachiya [17], when n = 7 and d = 3, R = 0.4844, and when n = 7 and d = 4, R = 0.3693. Using the method in this paper, the same encoding rate can be achieved with a shorter sequence, when n = 6 and d = 3, R = 0.4922, and when n = 6 and d = 4, R = 0.3962. This shows that the DNA storage coding set design model in this paper has better storage performance.

### 4.2. Lower Bound of Coding Set with Enhanced Constraints

In order to enhance the robustness of DNA storage coding set, this research combines enhanced constraints (RTSC, improved DTW distance) and traditional constraints (GC, NL) as new combined constraints; that is, Equation (10) is used to calculate the fitness function and constructs a new coding set according to the above construction steps. The set of DNA sequences whose length is n and the improved DTW distance is d_DTW_ is represented by S^GC,NL,RTSC^(n,d_DTW_), and the number of coding sets constructed by ROEAO with the above constraints is represented in Table 6.

In order to verify that the coding quality of S^GC,NL,RTSC^(n,d_DTW_) is improved with enhanced constraints, the chemical and physical properties of S^GC,NL^(n,d) and S^GC,NL,RTSC^(n,d_DTW_) were compared. The number of hairpin structures is one of the criteria for judging the stability of physical properties of a sequence. The melting temperature (Tm) refers to the temperature when the ultraviolet absorption of denatured nucleic acid reaches half of the maximum, and it is the main index to judge the chemical properties of DNA [45]. Therefore, this research used these two indicators to verify whether the quality of S^GC,NL,RTSC^(n,d_DTW_) is enhanced.

### 4.3. Comparison Results of Set Quality

#### 4.3.1. Hairpin Structures

Table 7 shows a comparison of the number of hairpin structures in the S^GC,NL^(n,d) and S^GC,NL,RTSC^(n,d_DTW_) collections. It can be seen from Table 7 that S^GC,NL,RTSC^(n,d_DTW_) had a smaller number of hairpins under different sequence lengths and different Hamming distances, indicating that the physical properties of the coding in S^GC,NL,RTSC^(n,d_DTW_) was improved.

Table 8 shows the comparison of the ratio of the number of hairpin structures to the number of DNA sequences in S^GC,NL^(n,d) and S^GC,NL,RTSC^(n,d_DTW_). The smaller the ratio is, the more stable the physical properties of the sequences are. The data in the table show that when the ratio was 8, the ratio was reduced by 1~41%; when the ratio was 9, the ratio was reduced by 2~23%; and when the ratio was 10, the ratio was reduced by 4~9%. It can be seen that the ratio of card issuing structure decreases in varying degrees, which proves that the enhanced constraint can bring more stable physical properties to the coding of DNA sequences.

#### 4.3.2. Melting Temperature

The comparison results of the Tm variances of S^GC,NL^(n,d) and S^GC,NL,RTSC,DTW^(n,d) are shown in Table 9. From the data in the table, when n equaled 8, 9, and 10, respectively, the variance of Tm decreased by 3~25%, 6~36%, and 3~68%, respectively; that is, each subset of S^GC,NL,RTSC,DTW^(n,d) had a more stable Tm performance. It is proved that the enhanced constraint can provide a more stable Tm value for DNA storage coding.

In summary, the DNA storage sets that meet the enhanced constraints have better data performance in the evaluation of the number of hairpin structures and the evaluation of the melting temperature, indicating that RTSC and improved DTW distance constraints bring better physical and chemical stability to DNA coding storage sets, which can effectively reduce the number of DNA secondary structures in the coding set, thus as to ensure the smooth progress of the reading phase in DNA storage.

## 5. Conclusions

To avoid errors in the reading phase while raising the lower bound of DNA storage coding sets, a method of building more stable DNA storage sets was proposed in this research. Firstly, in order to solve the issue of secondary structure caused by the tandem repetition of DNA sequences in the process of information coding, a repeated tandem sequence constraint was proposed. In order to address the issue that the traditional distance constraint is not accurate in evaluating the overall similarity between sequences, which cannot effectively limit the occurrence of non-specific hybridization reactions between them, an improved DTW distance constraint was proposed in this research, and through the comparison of biological simulation experiments and improved DTW analysis map, it was proved that the enhanced constraint could predict and limit the occurrence of a non-specific hybridization reaction. Secondly, on the basis of the AO algorithm, we adjusted its search direction and step size, and obtained a ROEAO algorithm with good optimization performance. Further, the mainstream benchmark function was used by this research to test and compare the ROEAO algorithm with seven other meta-heuristic algorithms to verify the optimization ability of the improved ROEAO algorithm. The comparison results show that ROEAO had advantages in accuracy and stability in the test results of 13 functions, the theoretical optimal value was reached in the test of 7 functions, the optimal value was reached in almost every test function, and the accuracy was 12~37 orders of magnitude higher than that of other algorithms in F5, F6, F12, and F13 tests, reflecting the fast optimization speed of ROEAO and good performance of jumping out of local optimization.

Finally, the ROEAO algorithm with excellent development and exploration ability was combined with traditional constraints and obtained the DNA storage coding set, which is 9~28% higher than the lower bound of the previous research set, which shows that the code set constructed by ROEAO has the ability to store more information at the same length. Further, in order to prove that the robustness of DNA storage coding sets is improved with enhanced constraints, the physical and chemical stability was evaluated by testing the number of hairpin structures and the stability of the melting temperature. With the enhanced constraint, the ratio generated by the hairpin structure of the sets was reduced by 1~41% compared with the coding set without enhanced constraint, and the variance of the melting temperature was reduced by 3~68%, indicating that higher robustness of DNA storage coding sets can be obtained with the enhanced constraint.

In the experiment, the improved DTW distance can calculate the distance more in line with the biological properties of DNA by warping the DNA sequence and then aligning it, thus more truly measuring the possibility of non-specific hybridization between sequences. However, in some special cases, when calculating the DTW distance, pathological alignment will occur between some sequences, resulting in a smaller distance than the actual distance. Therefore, in the next work, we will try to further optimize the improved DTW distance formula to propose a distance with a wider range of constraints and apply it to the design of DNA storage coding.

## Figures and Tables

**Table 1:** The meaning of superscript.

Superscript	Meaning
R	The result from ROEAO
EO	The result from EORS
A	The result from Altruistic
T	The result from ROEAO with Traditional constraints
E	The result from ROEAO with Enhanced constraints

**Table 2:** Unimodal test results, dimension = 30.

ID	Metric	ROEAO	AO	GWO	WOA	HHO	MPA	PSO	DE
F1	AVG	**0.00 × 10^0^**	2.32 × 10^−112^	1.25 × 10^−28^	5.67 × 10^−75^	1.88 × 10^−98^	3.45 × 10^−23^	1.43 × 10^4^	1.26 × 10^−4^
	STD	**0.00 × 10^0^**	1.48 × 10^−114^	2.01 × 10^−28^	1.67 × 10^−74^	1.98 × 10^−98^	4.54 × 10^−23^	2.01 × 10^3^	2.92 × 10^−5^
F2	AVG	**0.00 × 10^0^**	6.44 × 10^−54^	6.71 × 10^−17^	4.78 × 10^−49^	3.44 × 10^−48^	2.646 × 10^−13^	3.38 × 10^2^	4.83 × 10^−4^
	STD	**0.00 × 10^0^**	4.13 × 10^−53^	4.63 × 10^−17^	3.56 × 10^−48^	2.63 × 10^−48^	2.36 × 10^−13^	1.33 × 10^3^	6.06 × 10^−4^
F3	AVG	**0.00 × 10^0^**	4.35 × 10^−101^	5.23 × 10^−06^	4.57 × 10^4^	3.36 × 10^−68^	1.74 × 10^−4^	3.25 × 10^4^	3.46 × 10^4^
	STD	**0.00 × 10^0^**	9.90 × 10^−101^	1.58 × 10^−6^	2.02 × 10^4^	2.41 × 10^−67^	1.26 × 10^−4^	8.91 × 10^3^	5.57 × 10^3^
F4	AVG	**0.00 × 10^0^**	1.06 × 10^−53^	1.23 × 10^−6^	3.72 × 10^1^	2.23 × 10^−47^	2.84 × 10^−9^	5.31 × 10^1^	1.33 × 10^1^
	STD	**0.00 × 10^0^**	3.90 × 10^−52^	8.32 × 10^−7^	3.38 × 10^1^	5.24 × 10^−47^	2.27 × 10^−9^	2.75 × 10^0^	2.65 × 10^0^
F5	AVG	**2.63 × 10^−15^**	4.45 × 10^−3^	2.64 × 10^1^	3.01 × 10^1^	1.01 × 10^−2^	25.32 × 10^0^	2.96 × 10^7^	1.44 × 10^2^
	STD	**1.41 × 10^−14^**	5.58 × 10^−3^	7.53 × 10^−1^	2.97 × 10^−1^	1.19 × 10^−2^	6.74 × 10^−1^	6.83 × 10^6^	1.78 × 10^2^
F6	AVG	**1.72 × 10^−29^**	1.62 × 10^−4^	7.42 × 10^−1^	2.41 × 10^−1^	1.48 × 10^−4^	2.81 × 10^−8^	1.88 × 10^4^	7.24 × 10^−4^
	STD	**5.84 × 10^−29^**	1.53 × 10^−4^	3.48 × 10^−1^	1.74 × 10^−1^	1.42 × 10^−4^	1.66 × 10^−8^	2.89 × 10^3^	5.36 × 10^−4^
F7	AVG	1.55 × 10^−4^	**9.67 × 10^−5^**	1.68 × 10^−3^	1.75 × 10^−3^	1.31 × 10^−4^	1.33 × 10^−3^	1.27 × 10^1^	5.56 × 10^−2^
	STD	4.24 × 10^−4^	1.57 × 10^−4^	1.16 × 10^−3^	1.52 × 10^−3^	**1.62 × 10^−4^**	5.36 × 10^−4^	3.45 × 10^0^	1.84 × 10^−2^

**Table 3:** Multi-modal test results, dimension = 30.

ID	Metric	ROEAO	AO	GWO	WOA	HHO	MPA	PSO	DE
F8	AVG	**−1.29 × 10^4^**	−6.69 × 10^3^	−5.58 × 10^3^	−1.23 × 10^4^	−1.26 × 10^4^	−8.34 × 10^3^	−2.86 × 10^3^	−5.47 × 10^3^
	STD	6.27 × 10^3^	3.53 × 10^3^	7.10 × 10^2^	1.67 × 10^3^	**1.83 × 10^2^**	5.38 × 10^2^	2.79 × 10^2^	2.73 × 10^2^
F9	AVG	**0.00 × 10^0^**	**0.00 × 10^0^**	2.12 × 10^0^	1.36 × 10^−15^	**0.00 × 10^0^**	**0.00 × 10^0^**	1.87 × 10^2^	1.92 × 10^2^
	STD	**0.00 × 10^0^**	**0.00 × 10^0^**	3.09 × 10^0^	1.01 × 10^−14^	**0.00 × 10^0^**	**0.00 × 10^0^**	1.15 × 10^1^	1.94 × 10^1^
F10	AVG	**8.88 × 10^−16^**	**8.88 × 10^−16^**	1.23 × 10^−13^	3.57 × 10^−15^	**8.88 × 10^−16^**	2.95 × 10^−12^	1.71 × 10^1^	1.41 × 10^−2^
	STD	**0.00 × 10^0^**	**0.00 × 10^0^**	1.82 × 10^−14^	2.32 × 10^−15^	3.41 × 10^−31^	1.57 × 10^−12^	3.62 × 10^−1^	3.45 × 10^−3^
F11	AVG	**0.00 × 10^0^**	**0.00 × 10^0^**	2.56 × 10^−3^	5.56 × 10^−3^	**0.00 × 10^0^**	**0.00 × 10^0^**	1.72 × 10^2^	4.58 × 10^−2^
	STD	**0.00 × 10^0^**	**0.00 × 10^0^**	8.77 × 10^−3^	3.74 × 10^−2^	**0.00 × 10^0^**	**0.00 × 10^0^**	3.27 × 10^1^	7.12 × 10^−2^
F12	AVG	**1.41 × 10^−30^**	5.31 × 10^−6^	3.84 × 10^−2^	2.36 × 10^−2^	8.23 × 10^−6^	1.26 × 10^−5^	1.54 × 10^7^	1.35 × 10^−3^
	STD	**6.08 × 10^−30^**	7.26 × 10^−6^	1.92 × 10^−2^	2.65 × 10^−2^	1.72 × 10^−5^	6.68 × 10^−5^	9.78 × 10^6^	2.72 × 10^−3^
F13	AVG	**2.76 × 10^−29^**	2.53 × 10^−5^	6.16 × 10^−1^	5.76 × 10^−1^	2.11 × 10^−4^	1.66− × 10^−2^	5.53 × 10^7^	8.12 × 10^−3^
	STD	**1.13 × 10^−28^**	4.66 × 10^−5^	2.73 × 10^−1^	2.15 × 10^−1^	2.27 × 10^−4^	5.46 × 10^−2^	3.08 × 10^7^	2.74 × 10^−2^

**Table 4:** Wilcoxon statistical test results.

Comparison	s/e/w	*p*-Value
AO vs. ROEAO	9/3/1	3.6658 × 10^−2^
GWO vs. ROEAO	13/0/0	1.4740 × 10^−3^
WOA vs. ROEAO	13/0/0	1.4740 × 10^−3^
HHO vs. ROEAO	9/3/1	2.8417 × 10^−2^
MPA vs. ROEAO	11/2/0	3.3460 × 10^−3^
PSO vs. ROEAO	13/0/0	1.4740 × 10^−3^
DE vs. ROEAO	13/0/0	1.4740 × 10^−3^

**Table 5:** Lower bounds for S^GC,NL^(n,d).

n\d	3	4	5	6	7	8	9
4	11 ^A^						
	**12 ^EO^**						
	**12 ^R^**						
5	17 ^A^	7 ^A^					
	**20 ^EO^**	**8 ^EO^**					
	**20 ^R^**	**8 ^R^**					
6	44 ^A^	16 ^A^	6 ^A^				
	55 ^EO^	21 ^EO^	**8 ^EO^**				
	**60 ^R^**	**27 ^R^**	**8 ^R^**				
7	110 ^A^	36 ^A^	11^A^	4 ^A^			
	125 ^EO^	46 ^EO^	16 ^EO^	6 ^EO^			
	**127 ^R^**	**47 ^R^**	**17 ^R^**	**7 ^R^**			
8	289 ^A^	86 ^A^	29 ^A^	9 ^A^	4 ^A^		
	326 ^EO^	**110 ^EO^**	**38 ^EO^**	**15 ^EO^**	**5 ^EO^**		
	**327 ^R^**	**110 ^R^**	36 ^R^	14 ^R^	**5 ^R^**		
9	662 ^A^	199 ^A^	59 ^A^	15 ^A^	8 ^A^		
	737 ^EO^	226 ^EO^	**71 ^EO^**	26 ^EO^	**11 ^EO^**	**5 ^EO^**	
	**786^R^**	**228^R^**	**71^R^**	**27 ^R^**	**11^R^**	**5 ^R^**	
10	1810 ^A^	525 ^A^	141 ^A^	43 ^A^	7 ^A^	5 ^A^	4 ^A^
	1856 ^EO^	546 ^EO^	153 ^EO^	53 ^EO^	**22 ^EO^**	9 ^EO^	**5 ^EO^**
	**1964 ^R^**	**581^R^**	**157 ^R^**	**57 ^R^**	21^R^	**10 ^R^**	**5 ^R^**

**Table 6:** Lower bounds for S^GC,NL,RTSC^(n,d_DTW_).

n/d_DTW_	3	4	5	6	7	8	9
8	170 ^E^	40 ^E^	14 ^E^	5 ^E^	3 ^E^		
9	314 ^E^	83 ^E^	21 ^E^	8 ^E^	4 ^E^	2 ^E^	
10	607 ^E^	155 ^E^	34 ^E^	11 ^E^	6 ^E^	3 ^E^	1 ^E^

**Table 7:** Comparison of the number of hairpin structures between S^GC,NL^(n,d) and S^GC,NL,RTSC^(n,d_DTW_).

n/d	3	4	5	6	7	8	9
8	170 ^T^	40 ^T^	14 ^T^	5 ^T^	3 ^T^		
	**67 ^E^**	**16 ^E^**	**5 ^E^**	**2 ^E^**	**1 ^E^**		
9	403 ^T^	112 ^T^	32 ^T^	11 ^T^	6 ^T^	4 ^T^	
	**314 ^E^**	**83 ^E^**	**21 ^E^**	**8 ^E^**	**4 ^E^**	**2 ^E^**	
10	1776 ^T^	442 ^T^	100 ^T^	33 ^T^	14 ^T^	8 ^T^	2 ^T^
	**607 ^E^**	**155 ^E^**	**34 ^E^**	**11 ^E^**	**6 ^E^**	**3 ^E^**	**1^E^**

**Table 8:** Comparison of the ratio of hairpin structure between S^GC,NL^(n,d) and S^GC,NL,RTSC^(n,d_DTW_).

n/d	3	4	5	6	7	8	9
8	0.4709 ^T^	0.4273 ^T^	0.3611 ^T^	0.5714 ^T^	0.4000 ^T^		
	**0.3941 ^E^**	**0.4000 ^E^**	**0.3571 ^E^**	**0.3333 ^E^**	**0.3333 ^E^**		
9	1.4389 ^T^	1.4430 ^T^	1.5775 ^T^	1.4074 ^T^	1.5455 ^T^	2.6000 ^T^	
	**1.2834 ^E^**	**1.3494 ^E^**	**1.5238 ^E^**	**1.3750 ^E^**	**1.5000 ^E^**	**2.0000 ^E^**	
10	3.0351 ^T^	3.0637 ^T^	3.1210 ^T^	3.2807 ^T^	2.4762 ^T^	2.8000 ^T^	**2.0000 ^T^**
	**2.9259 ^E^**	**2.8516 ^E^**	**2.9412 ^E^**	**3.0000 ^E^**	**2.3333 ^E^**	**2.6667 ^E^**	**2.0000 ^E^**

**Table 9:** Comparison of Tm variance between S^GC,NL^(n,d) and S^GC,NL,RTSC^(n,d_dtw_).

n/d	3	4	5	6	7	8	9
8	5.913 ^T^	6.5933 ^T^	6.1047 ^T^	3.7399 ^T^	3.0728 ^T^		
	**5.6656 ^E^**	**6.4030 ^E^**	**4.5663 ^E^**	**3.5999 ^E^**	**2.3000 ^E^**		
9	5.1303 ^T^	5.0506 ^T^	5.1692 ^T^	6.2964 ^T^	2.9481 ^T^	3.5663 ^T^	
	**4.8113 ^E^**	**4.4362 ^E^**	**4.3375 ^E^**	**4.0537 ^E^**	**2.5743 ^E^**	**2.3743 ^E^**	
10	4.7194 ^T^	4.5276 ^T^	5.1658 ^T^	4.7232 ^T^	3.5348 ^T^	3.3421 ^T^	1.6232 ^T^
	**4.5559 ^E^**	**4.3101 ^E^**	**4.3337 ^E^**	**3.5932 ^E^**	**2.8485 ^E^**	**2.9237 ^E^**	**0.5121 ^E^**

## Data Availability

The data that support the findings of this study are available from UCI Machine Learning Repository. Restrictions apply to the availability of these data, which were used under license for this study.
